# ASSOCIATIONS BETWEEN LONELINESS AND COGNITIVE RESILIENCE TO NEUROPATHOLOGY IN OLDER ADULTS

**DOI:** 10.1093/geroni/igae098.1350

**Published:** 2024-12-31

**Authors:** Kathryn Jackson

**Affiliations:** Northwestern University, Evanston, Illinois, United States

## Abstract

Loneliness in the aging population is associated with decreased cognitive function and increased neuropathology; less is understood, however, about the association of loneliness and cognitive resilience (CR), defined as the discordance between a person’s actual and expected cognition given their neuropathology. For this study, data were combined from 2 longitudinal studies of older adults. CR proximate to death (CRlast_level) and across time (CRslope) were obtained by independently regressing global cognition and change in cognition onto multiple neuropathology indicators and extracting the resulting residuals. Using a series of linear regression models, we assessed the effect of loneliness level and change in loneliness on CR. Results show that higher and increasing loneliness was associated with lower CR in the face of neuropathology, suggesting that some individuals are less resilient to the accumulation of neuropathology than others, and experiencing high/increasing loneliness is a key factor putting some at risk.

